# Metagenome-assembled genomes from microbial communities producing lactic acid from dairy residues

**DOI:** 10.1128/mra.00179-25

**Published:** 2025-04-29

**Authors:** Faith Koester, Kevin S. Myers, Timothy J. Donohue, Daniel R. Noguera

**Affiliations:** 1 Wisconsin Energy Institute and Great Lakes Bioenergy Research Center, University of Wisconsin-Madison 732059, Madison, Wisconsin, USA; 2 Department of Bacteriology, University of Wisconsin-Madison 205263, Madison, Wisconsin, USA; 3 Department of Civil and Environmental Engineering, University of Wisconsin-Madison 539348, Madison, Wisconsin, USA; Montana State University, Bozeman, Montana, USA

**Keywords:** metagenomics, dairy residues, lactic acid, microbial communities

## Abstract

To advance the knowledge of microbial communities capable of fermenting agro-industrial residues into value-added products, we report metagenomes of microbial communities from four anaerobic bioreactors fed a mixture of ultra-filtered milk permeate and cottage cheese acid whey. This analysis produced 42 unique metagenome-assembled genomes (MAGs) that represent distinct taxa.

## ANNOUNCEMENT

Microbial communities have the potential to increase the value of agro-industrial residues by fermentation. Metagenomes reported here originate from two sets of anaerobic bioreactors and their inoculant cultures. In each experiment, two chemostats were operated anaerobically, at 50°C, pH 5.5, 6-day solids retention time, and either a 6-, 3-, or 1-day hydraulic retention time. The chemostats were supplied with a 1:1 mixture of ultra-filtered milk permeate and cottage cheese acid whey and inoculated with a culture derived from an acid-phase digester at a wastewater treatment plant (Madison, WI, USA) that was first enriched in a similarly operated bioreactor for 26 days at 50°C.

For each experiment, weekly samples of DNA were extracted from the microbes in each bioreactor with the Qiagen Genomic Tip 20 /G (Qiagen) following the manufacturer’s protocol for “Preparation Gram‐negative and some Gram‐positive Bacterial Samples” with lysostaphin (4000 U mL^−1^, Sigma‐Aldrich) added. A total of 37 samples containing 3,000–10,000 ng DNA were sequenced on a Pacific Biosciences Sequel 2 platform by the Joint Genome Institute (JGI) (Berkeley, USA).

Processing and sequencing of DNA included shearing genomic DNA to either 3 kb or 6–10 kb, depending on DNA quality, and performing ligations using the SMRTbell Express template prep kit (Pacific Biosciences). Size selection was performed with BluePippin (Sage Sciences). Finally, the libraries were sequenced on Sequel II (Pacific Biosciences, Inc. [PacBio], Menlo Park, CA, USA) using Sequel Polymerase Binding Kit and Revio chemistry. Across all 37 samples, there was an average of 513,455 reads with a range from 341,988 reads to 828,421 reads. The average length was 9,120 bp (range of 8,037 bp to 10,220 bp), and the average N50 of the reads was 9,367 bp (range of 8,033 bp to 10,531 bp).

Metagenomic DNA quality checking, assembly, binning, and taxonomy classification were performed as follows, and default parameters were used except where otherwise noted. BBTools (v39.01) (1) was used to remove reads containing inverted repeats. Followed by assembly with MetaMDBG (v0.3) (2) (“metaMDBG asm assembly <fastq.gz> -t 4”), polished with Pbmm2 (v1.13.1) (3) and Racon (v1.5.0) (4) for consensus, aligned with minimap2 (v2.26-r1175) (5). Then, “pileup.sh” from BBTools (1) to calculate coverage and binning with MetaBAT (v2.15) (6). Quality was assessed with checkM (v1.2.2) (7), and MAGs were dereplicated using dRep (v3.4.5) (-N50W 2) (8). GTDB-Tk (v2.3.2, database release 214) (9) was used to assign taxonomy to representative MAGs (“gtdbtk identify”) based on concatenated bacterial marker genes (Bac120). For NCBI submission, some taxonomic changes were required as noted in Table 1. Bakta (v1.9.1) (10) was used for gene annotation of all MAGs.

**TABLE 1:** Taxonomy, accession numbers, genomic features, and quality statistics of the 42 high- and medium-quality representative MAGs recovered from the bioreactors

MAG ID	Phylum	GTDB classification^*a*^	NCBI classification^*b*^	Reference genome^*c*^	ANI^*d*^	Completeness (%)	Contamination (%)	MAG size (bp)	Contigs	N50 (bp)	%GC	NCBI accession number	NCBI SRA accession number^*e*^
PSEUD1	Pseudomonadota	*Pseudomonas_E helleri*	*Pseudomonas helleri*	GCF_001043025.1	97.28	100	0.63	5,950,624	1	5,950,624	58.07	CP181271	SRR31045131
BIF1	Actinomycetota	*Bifidobacterium crudilactis*	*Bifidobacterium crudilactis*	GCF_000738005.1	97.36	99.08	2.12	2,454,116	1	2,454,116	57.86	CP181270	SRR31045102
LENTI1	Bacillota	*Lentilactobacillus parabuchneri*	*Lentilactobacillus parabuchneri*	GCF_001435315.1	97.43	98.94	0	2,574,352	1	2,574,352	43.68	CP181269	SRR31045178
LACTICAS1	Bacillota	*Lacticaseibacillus paracasei*	*Lacticaseibacillus casei*	GCF_000829035.1	98.43	98.64	2.47	2,927,657	29	303,583	46.58	JBKEEX000000000	SRR31045161
LEUC1	Bacillota	*Leuconostoc mesenteroides*	*Leuconostoc mesenteroides*	GCF_000014445.1	98.78	91.8	0	1,925,562	8	1,350,794	37.81	JBKEEW000000000	SRR31045102
CLOS1	Bacillota A	*Clostridium_H*	*Clostridium*	N/A	N/A	94.71	7.62	2,653,518	18	408,933	31.39	JBKEEV000000000	SRR31045122
CLOS2	Bacillota A	*Clostridium_B sp014050525*	*Clostridium*	GCF_014050525.1	99.37	99.59	0	2,617,478	1	2,617,478	30.11	CP181268	SRR31045172
THERMO1	Bacillota A	*Thermoanaerobacterium thermosaccharolyticum*	*Thermoanaerobacterium thermosaccharolyticum*	GCF_000145615.1	97.17	99.4	4.11	3,240,700	12	2,846,837	34.01	JBKEEU000000000	SRR31045111
SUTT1	Pseudomonadota	*Sutterella*	*Burkholderia*	N/A	N/A	96.89	0	2,299,283	13	427,523	63.29	JBKEET000000000	SRR31045162
ATO1	Actinomycetota	*UBA7748 sp900314535*	*Atopobium*	GCA_900314535.1	96.94	99.19	0.81	2,622,559	2	2,593,049	60.25	JBKEES000000000	SRR31045112
LACTIPLAN1	Bacillota	*Lactiplantibacillus plantarum*	*Lactiplantibacillus plantarum*	GCF_014131735.1	96.07	99.38	2.78	3,078,116	2	2,813,036	45.26	JBKEER000000000	SRR31045116
PSEUD2	Pseudomonadota	*Pseudomonas_E fragi*	*Pseudomonas fragi*	GCF_900105835.1	98.55	86.74	0.27	4,465,640	40	177,538	59.39	JBKEEQ000000000	SRR31045170
LACTO1	Bacillota	*Lactobacillus delbrueckii*	*Lactobacillus delbrueckii*	GCF_001433875.1	97.69	99.03	0	2,184,090	1	2,184,090	49.14	CP181267	SRR31045170
LACTO2	Bacillota	*Lactobacillus helveticus*	*Lactobacillus helveticus*	GCF_000160855.1	97.68	99.03	0.32	2,234,393	1	2,234,393	36.92	CP181266	SRR31045189
LIMO1	Bacillota	*Limosilactobacillus fermentum*	*Limosilactobacillus fermentum*	GCF_013394085.1	98.91	96.86	0	2,025,386	14	154,414	51.82	JBKEEP000000000	SRR31045178
SUCC1	Bacillota C	*Succiniclasticum*	*Succiniclasticum*	N/A	N/A	98.58	0	2,160,246	1	2,160,246	55.98	CP181265	SRR31045174
ACID1	Bacillota C	*Acidaminococcus timonensis*	*Acidaminococcus timonensis*	GCF_900106585.1	96.53	99.4	0	2,286,539	1	2,286,539	54.95	CP181264	SRR31045164
CAPRO1	Bacillota A	*Caproiciproducens sp012516295*	*Eubacteriales*	GCA_012516295.1	99.7	97.99	0.27	2,530,718	1	2,530,718	48.71	CP181263	SRR31045122
CAPRO2	Bacillota A	*Caproicibacter sp024498295*	*Thermocaproicibacter melissae*	GCA_024498295.1	99.27	97.32	0	2,007,289	1	2,007,289	49.47	CP181262	SRR31045122
CAPRO3	Bacillota A	*Caproicibacter*	*Clostridium*	N/A	N/A	97.99	0	2,650,324	1	2,650,324	44.45	CP181261	SRR31045102
CAPRO4	Bacillota A	*Caproicibacter*	*Oscillospiraceae*	N/A	N/A	97.99	0.67	2,802,872	6	1,505,020	45.14	JBKEEO000000000	SRR31045122
LACTOCO1	Bacillota	*Lactococcus lactis*	*Lactococcus lactic*	GCF_900099625.1	98.52	100	0.28	2,595,605	1	2,595,605	35.1	CP181260	SRR31045131
PSEUD3	Pseudomonadota	*Pseudomonas_E lundensis*	*Pseudomonas lundensis*	GCF_001042985.1	98.05	94.32	0.11	4,386,151	7	913,462	58.66	JBKEEN000000000	SRR31045166
LACTOCO2	Bacillota	*Lactococcus_A*	*Lactococcus*	N/A	N/A	98.11	0.5	2,242,213	1	2,242,213	41.54	CP181259	SRR31045116
LACTOCO3	Bacillota	*Lactococcus_A raffinolactis*	*Lactococcus raffinolactis*	GCF_001591765.1	98.66	98.11	0.72	2,239,778	1	2,239,778	39.97	CP181258	SRR31045156
VIRG1	Bacillota	*Virgibacillus proomii_A*	*Virgibacillus proomii*	GCF_900162615.1	99.58	100	1.67	4,423,115	10	1,262,884	36.16	JBKEEM000000000	SRR31045166
ENT1	Bacillota	*Enterococcus_A gilvus*	*Enterococcus gilvus*	GCF_000407545.1	97.95	98.85	3.4	3,781,005	5	2,660,674	42.13	JBKEEL000000000	SRR31045166
ENT2	Bacillota	*Enterococcus_B faecium*	*Entercococcus faecium*	GCF_001544255.1	98.62	99.63	0	2,753,251	1	2,753,251	38.16	CP181257	SRR31045113
DIAL1	Bacillota C	*Dialister sp900765565*	*Dialister*	GCA_900765565.1	96.99	99.59	0	2,944,120	1	2,944,120	51.49	CP181256	SRR31045111
MEGA1	Bacillota C	*Megasphaera sp000417505*	*Megasphaera*	GCF_000417505.1	98.54	100	0	2,329,985	1	2,329,985	54.02	CP181255	SRR31045111
SELE1	Bacillota C	*Selenomonas_C sp934202005*	*Selenomonas*	GCA_934202005.1	95.96	100	0	2,276,171	1	2,276,171	60.17	CP181254	SRR31045116
MITSU1	Bacillota C	*Mitsuokella sp900552565*	*Mitsuokella multacida*	GCA_900552565.1	95.27	99.07	0.14	2,353,814	1	2,353,814	53.79	CP181253	SRR31045122
XYLA1	Bacillota A	*Xylanivirga thermophila*	*Xylanivirga thermophila*	GCF_004138105.1	98.99	97.58	1.21	2,650,085	14	339,090	36.41	JBKEEK000000000	SRR31045122
SPOR1	Bacillota A	*Sporanaerobacter*	*Sporanaerobacter*	N/A	N/A	99.3	0.7	2,267,806	1	2,267,806	31.32	CP181252	SRR31045122
PSEUD4	Pseudomonadota	*Pseudomonas_E shahriarae*	*Pseudomonas shahriarae*	GCF_014268455.2	99.27	99.32	1.21	6,278,246	1	6,278,246	60.55	CP181251	SRR31045170
BACI1	Bacillota	*Bacillus_H clausii*	*Shouchella clausii*	GCF_002250115.1	97.89	98.67	2	4,672,023	1	4,672,023	44.46	CP181250	SRR31045113
ANA1	Bacillota A	*Anaerocolumna*	*Lachnoclostridium*	N/A	N/A	94.62	1.57	3,467,848	49	146,365	37.8	JBKEEJ000000000	SRR31045122
HAF1	Pseudomonadota	*Hafnia paralvei*	*Hafnia paralvei*	GCF_001655005.1	99.06	99.86	0.45	4,969,061	1	4,969,061	48.1	CP181249	SRR31045104
SERR1	Pseudomonadota	*Serratia_J liquefaciens*	*Serratia liquefaciens*	GCF_000422085.1	98.86	100	0.45	5,585,869	1	5,585,869	55.23	CP181248	SRR31045173
RAHN1	Pseudomonadota	*Rahnella inusitata*	*Rahnella inusitata*	GCF_003263515.1	98.97	99.84	0.08	5,186,034	4	3,490,194	53	JBKEEI000000000	SRR31045170
CITRO1	Pseudomonadota	*Citrobacter braakii*	*Citrobacter braakii*	GCF_002075345.1	98.53	98.21	0.56	5,116,397	30	324,668	52.12	JBKEEH000000000	SRR31045122
PSEUDOCLA1	Actinomycetota	*Pseudoclavibacter_A caeni*	*Pseudoclavibacter caeni*	GCF_008831125.1	99.16	98.07	0	2,066,244	5	507,308	71.16	JBKEEG000000000	SRR31045165

^*a*^ Classification from GTDB-Tk (data release 214) (9) to the most defined taxonomic level.

^*b*^ Classification from NCBI to the most defined taxonomic level.

^*c*^ NCBI Genome Accession number for the closest genome as determined by GTDB-Tk (9). This is used to compare the MAG for ANI calculation by GTDB-Tk (9). N/A indicates that GTDB-Tk was unable to identify a reference genome that fell within the default parameters.

^*d*^ ANI (Average Nucleotide Identity) was determined from GTDB-Tk (9) using default parameters. N/A indicates that GTDB-Tk was unable to identify a reference genome that fell within the default parameters and so no ANI was calculated.

^*e*^ NCBI SRA Accession Number for the sample from which the MAG was identified.

This announcement reports 42 annotated MAGs that are classified as high- and medium-quality according to Bowers et al. (11), dereplicated and representing distinct taxa (Table 1). These data advance our metagenome-based knowledge of agro-industrial residue and waste bioconverting microbiomes.

## Data Availability

Raw metagenomic sequencing data are available at the following NCBI SRA Accession Numbers: SRR31045102, SRR31045104, SRR31045108, SRR31045109, SRR31045111, SRR31045112, SRR31045113, SRR31045114, SRR31045115, SRR31045116, SRR31045122, SRR31045124, SRR31045131, SRR31045133, SRR31045134, SRR31045156, SRR31045157, SRR31045161, SRR31045162, SRR31045163, SRR31045164, SRR31045165, SRR31045166, SRR31045167, SRR31045168, SRR31045169, SRR31045170, SRR31045171, SRR31045172, SRR31045173, SRR31045174, SRR31045175, SRR31045176, SRR31045177, SRR31045178, SRR31045189, SRR31045217. All MAG data are available at NCBI GenBank under BioProject ID PRJNA1159295. Individual NCBI genome accession numbers for all 42 MAGs are displayed in Table 1. All custom scripts are available on GitHub (https://github.com/GLBRC/metagenome_analysis).
